# Comparison of Metagenomic Sequencing and the NanoString nCounter Analysis System for the Characterization of Bacterial and Viral Communities in Vaginal Samples

**DOI:** 10.1128/msphere.00197-22

**Published:** 2022-08-24

**Authors:** Todd N. Wylie, Jane Schrimpf, Haley Gula, Brandi N. Herter, Kristine M. Wylie

**Affiliations:** a Department of Pediatrics, Washington University School of Medicine, St. Louis, Missouri, USA; b McDonnell Genome Institute, Washington University School of Medicine, St. Louis, Missouri, USA; CDC

**Keywords:** NanoString nCounter, microbiome, sequencing, vagina, virome

## Abstract

DNA sequencing assays have been used to characterize the vaginal microbiome and to identify associations with clinical outcomes. The purpose of this study was to evaluate the utility of the NanoString nCounter platform, a more efficient assay compared to sequencing, for the characterization of vaginal microbial communities. A panel of NanoString nCounter probes was designed to detect common vaginal bacteria and viruses with relevance to reproductive health. A defined synthetic community of microbes and 43 clinical samples were interrogated with NanoString nCounter assays and compared to known compositions or metagenomic shotgun sequencing (MSS) results. The NanoString nCounter platform and MSS were able to distinguish closely related microbes. In clinical samples, the relative abundance of bacterial species was similar between the two assays. The assays sometimes disagreed when targets were present at low abundance. More viruses were detected by MSS than by nCounter. However, the nCounter assays are able to provide results in about 30 h with minimal hands-on time, whereas MSS requires at least 138 to 178 h with extensive hands-on time. The reagent cost for the two assays was similar, but the overall cost of the nCounter was lower due to the minimal hands-on time. MSS can be used to inform the design of a targeted multiplex panel for the assessment of vaginal microbial communities, thereby allowing for more cost-effective and rapid screening of patient samples for research studies. The sensitivity for low abundance microbes could be improved, possibly by adding additional target amplification cycles before nCounter assessment. This approach has potential as an assay with both research and clinical applications.

**IMPORTANCE** Metagenomic shotgun sequencing can inform the design of a targeted multiplex panel by which the NanoString nCounter platform can assess vaginal microbial communities, thereby allowing for more cost-effective and rapid screening of patient samples.

## INTRODUCTION

Many studies have demonstrated associations between the composition and traits of microbial communities with clinical outcomes. For example, characteristics of the bacterial communities in the vagina have been associated with preterm birth, poor *in vitro* fertilization outcomes, bacterial vaginosis, and other conditions (1–11). High throughput sequencing-based assays have been instrumental in studies that have demonstrated associations between microbes and clinical conditions (12–14). Sequencing assays allow us to look at microbial communities broadly and without preconceptions about which features might be important for health or disease states. This is useful for examining both specific taxa and community characteristics (e.g., diversity, stability) that may be biologically relevant. Like other molecular assays, an advantage of sequencing is that it does not require culture. So, sequencing allows us to assess microbes that are difficult to culture or for which culturing conditions are unknown. While sequencing can be enormously advantageous for exploratory studies and for studies that build our understanding of microbial communities and their dynamics, it can be prohibitive in terms of both cost and time. Therefore, once communities are well-characterized, some studies may be better served by returning to well-designed, targeted molecular assays that capture the essential community characteristics relevant to the disease being studied. The development of simplified assays facilitates research and is a necessary step toward using assays of microbial community features as biomarkers for clinical conditions.

The NanoString nCounter System is a platform that can be used to rapidly and simultaneously assess up to 800 multiplexed targets with a sensitivity comparable to that of polymerase chain reaction (PCR) (15). Nucleic acid probe sequences are designed to bind to targets of interest, and each probe is tagged with a unique, fluorescent barcode. The barcodes are digitally detected and counted, yielding count data for each of the targets. This platform can be applied to generate counts for DNA or RNA molecules within a sample with a linear dynamic range of over 500-fold (15). Further, the hands-on time required for both the sample preparation and the analysis is minimal, making it a favorable option compared to more complex sequencing assays.

In this study, we demonstrate that the NanoString nCounter System can be used with a custom multiplex panel of bacterial and viral probes to characterize important features of vaginal microbial communities that have been established using sequencing assays. Compared with sequencing assays, the nCounter assay reduces costs, provides data in less time, and requires less intensive data production and analysis, making the nCounter advantageous for studies aimed at the rapid characterization of community composition.

## RESULTS

### Mock community.

**FIG 1:**
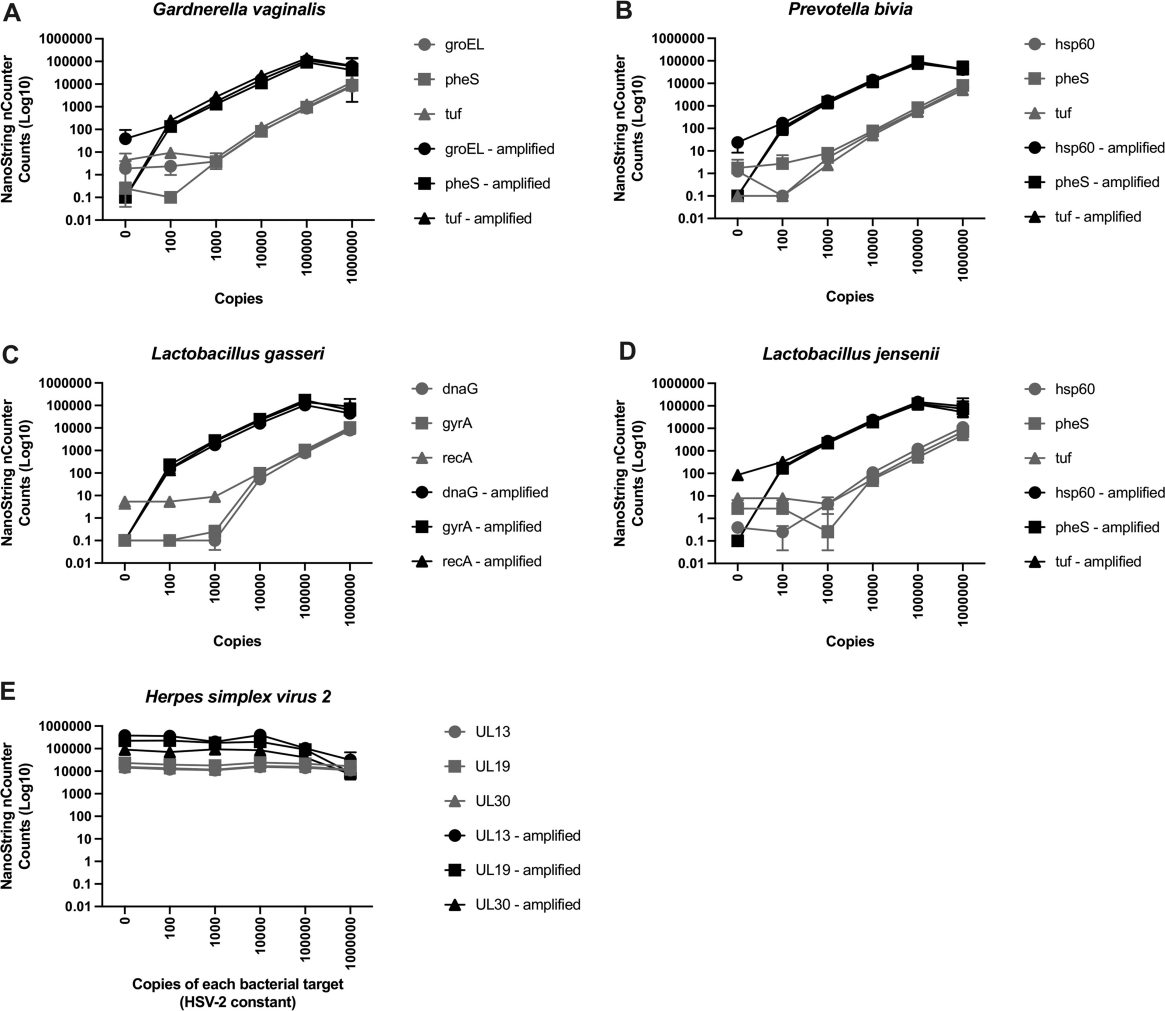
NanoString nCounter performance on a mock community. Panels A to E show data for different microbes included in the mock community. The *x* axis represents the number of copies of the target in the assay. The *y* axis represents the nCounter count for each probe. The nCounter assay without amplification is shown in light gray, and the assay with 8 cycles of amplification is shown in black. Each line represents data from a different gene target (3 genes per microbe). Data are from one representative experiment. Each point represents the mean from duplicate measures, and the error bars represent the standard deviation of the duplicates.

For our first experiments, we used serial dilutions of a mock community to test our ability to detect nucleic acid targets with the NanoString nCounter assay (Fig. 1A–E). We found that testing the samples directly (without target amplification) was insufficient for detecting 100 copies per reaction with confidence. We then tested the protocol with 8 cycles of PCR amplification prior to detection, and this greatly improved the detection, with 100 copies of each target being consistently detected. With 8 cycles of amplification, the nCounter counts from serial 10-fold dilutions of the targets yielded a linear curve, although at the highest concentration, the signal was dampened due to oversaturation. With 8 cycles of PCR amplification, the signals from the clinical samples with higher copy numbers oversaturated the nCounter system (not shown), so we reduced the number of cycles to 4 for testing clinical samples.

We also evaluated the reproducibility of our NanoString nCounter assay. Over 6 replicates, we found consistent counts detected by the probes (representative microbe, herpes simplex virus type 2 [HSV-2], shown in Fig. 1E). In the case shown, we found that all 3 independent HSV-2 probes were both reproducible and highly consistent with each other in the unamplified samples. With 8 cycles of amplification, the probes were still highly reproducible between experiments. When the 8 cycles of amplification were added, one probe had a slightly lower signal overall, compared to the other two probes, suggesting that the probe was not amplified as efficiently as the other two. However, all targets were amplified, as evidenced by comparisons with the unamplified targets.

### Mock community selectivity.

**FIG 2:**
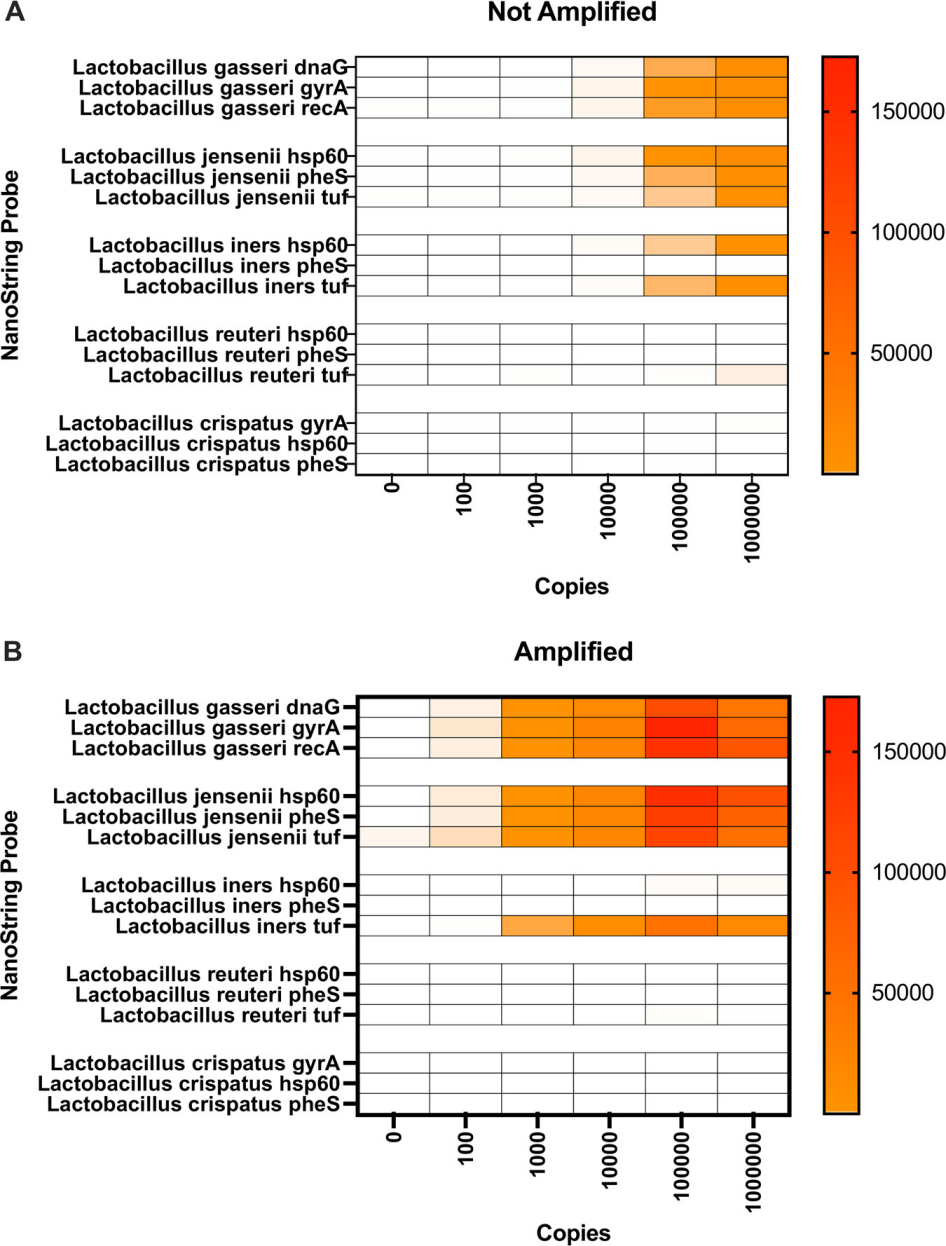
NanoString nCounter distinguishes *Lactobacillus* species. Panel A shows data from the assay in which the sample was not amplified, and Panel B shows data from the assay with 8 cycles of amplification. The *x* axis shows the number of copies of each target. The *y* axis shows each of 3 bacterial target genes for each of 5 *Lactobacillus* species. The light gray color indicates that no signal was detected, and an increasing intensity of orange to red represents increasing nCounter counts.

Next, we evaluated the selectivity of the probes among closely related taxa (Fig. 2). The ability to resolve taxa to the species level is important in understanding the microbial communities of the vagina. For example, the proper identification of the species of *Lactobacillus* in the vagina is critical, as different species are associated with different biological effects (16). Therefore, in our pilot study, we designed probes against 5 species of *Lactobacillus* and tested them on a mock community containing only 2 of those species. We found that the probes were able to detect the two species included in the mock community with the appropriate probes, while related *Lactobacillus* species were not detected. In the unamplified samples (Fig. 2A), 2 of the 3 Lactobacillus iners probes were cross-reactive; however, because we require all 3 probes to be positive, we were still able to discriminate the species. The additional selectivity added by the PCR primers during amplification helped to minimize the cross-reactivity of the individual probes such that only 1 of the 3 Lactobacillus iners probes was cross-reactive in the amplified condition.

### Clinical samples.

**FIG 3:**
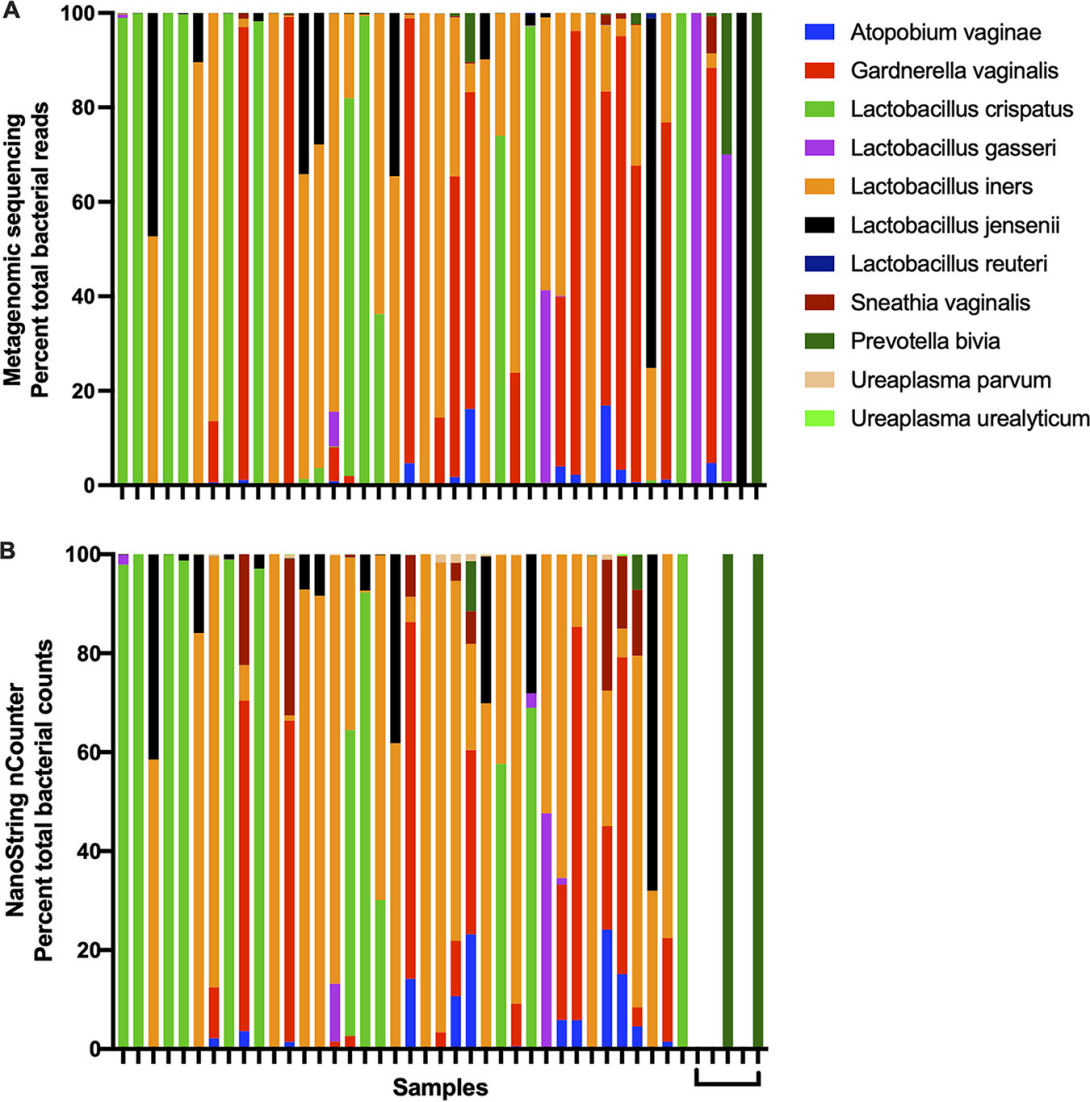
NanoString nCounter reproduces the relative abundance of bacterial communities. Panel A shows the relative abundance of bacterial taxa identified by metagenomic sequencing, and Panel B shows the results from the same samples evaluated by NanoString nCounter with 4 cycles of amplification. Each color represents a different taxon, listed in the key on the right. The different samples are indicated on the *x* axis. The percentage of bacterial reads is indicated on the *y* axis.

We tested our NanoString nCounter panel on 43 clinical vaginal swab samples to determine how the assay compared to sequencing results. The relative abundance of bacteria defined by metagenomic shotgun sequencing was largely reproduced by the nCounter assay (compare Fig. 3A and Fig. 3B). The last 5 samples on the right side of the figure showed poorer correlation between sequencing and the nCounter, with two samples not being detected by the nCounter. These 5 samples had low read counts supporting the bacteria identified (range of 455 to 12,061 reads in the metagenomic shotgun data). The agreement between the sequencing and nCounter assays varied among taxa, with the greatest agreement values found for Lactobacillus iners, Lactobacillus crispatus, and Gardnerella vaginalis (Cohen’s kappa values of 0.789, 0.890, and 0.856, respectively, indicating substantial to almost perfect agreement). However, some bacterial taxa were not detected frequently enough to adequately assess (e.g., L. reuteri). Others showed relatively poorer agreement. For example, Sneathia vaginalis was detected more frequently with the nCounter assay than with sequencing (Cohen’s kappa value of 0.382, indicating fair agreement), and the relative abundance was also higher in the nCounter assay compared with sequencing when *S. vaginalis* was detected. Overall, our data demonstrate that important community features that rely on relative abundance ratios of taxa (for example, the proportion of protective *Lactobacillus* species and some alpha diversity metrics), can be approximated by the nCounter assay and used in the evaluation of vaginal microbial communities (2–6).

**FIG 4:**
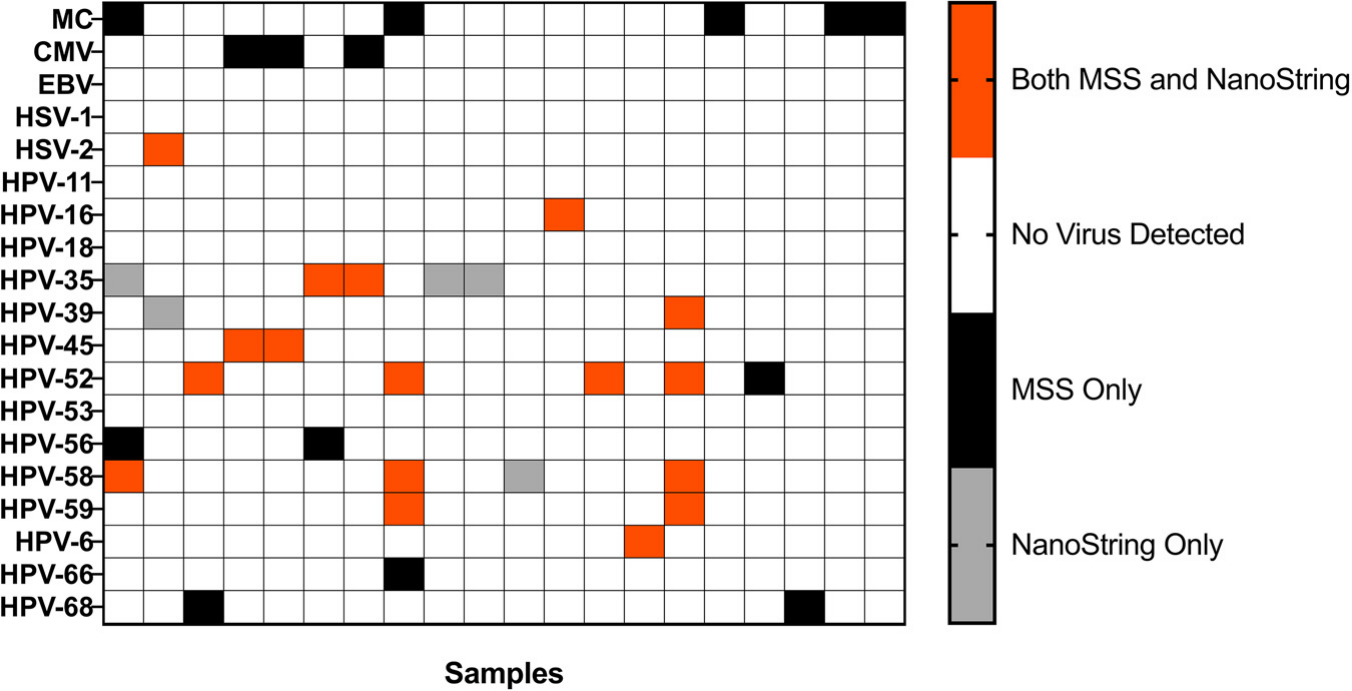
NanoString distinguishes viral species and types but detects fewer viruses than ViroCap with metagenomic shotgun sequencing. The different samples are indicated on the *x* axis. The different viruses are indicated on the *y* axis. The color of the box indicates which assay detected the virus (orange, both MSS and Nanostring; black, MSS only; gray, Nanostring only). MSS, metagenomic shotgun sequencing; MC, molluscum contagiosum; CMV, cytomegalovirus; EBV, Epstein-Barr virus; HSV, herpes simplex virus; HPV, human papillomavirus.

Finally, our group is especially well-poised to evaluate eukaryotic viruses in vaginal samples. We previously developed ViroCap, a targeted sequence capture panel that enriches the complete viral genomes from all known vertebrate viruses prior to metagenomic shotgun sequencing (17). ViroCap greatly improves virus detection in sequencing assays, making it comparable to sensitive PCR assays (17, 18). Our standard method for the analysis of the vaginal virome is ViroCap and sequencing (1). When we compared the NanoString nCounter to sequencing results from vaginal swabs from pregnant women, we found excellent resolution to the species/type level in both assays (Fig. 4). The NanoString nCounter results were also highly selective, with distinction between closely related viruses (HSV-1 and HSV-2; many types of human papilloma virus). Comparing the two assays, Cohen’s kappa was 0.63, indicating substantial agreement. Of the detected viruses, 17 out of 36 were found by both assays. We did find, however, that even with 4 cycles of amplification, the ViroCap sequencing assays were in some cases more sensitive than the nCounter assays (14 out of 36 detected viruses). Some viruses were detected by sequencing but were not detected at all by nCounter, including molluscum contagiosum and human cytomegalovirus (Fig. 4). In some samples (5 out of 36 detected viruses), viruses such as HPVs 58 and 35 were identified by nCounter but not by sequencing; however, we detected those viruses by both assays in other samples. A disagreement between methods only happened at low levels of virus, as estimated based on the low numbers of sequence reads detected or low nCounter counts.

### Time to results and cost.

We compared the time required and costs for reagents associated with the metagenomic sequencing and NanoString nCounter assays, based on our experiences (Table 1). Metagenomic sequencing required more time for sample preparation, data generation, and data analysis, compared with the nCounter assay. Sequencing with ViroCap enrichment took >138 h and included some resource-intensive and labor-intensive steps (e.g., sequencing library construction, computational analysis of the sequence data). In contrast, the nCounter assay took 30 h from DNA to report, requiring <1 h of hands-on time. The costs of reagents for the two assays were similar; however, when accounting for hands-on personnel time, sequencing becomes more expensive, overall.

**TABLE 1:** Comparison of time and cost associated with metagenomic sequencing and Nanostring nCounter assays

Time/cost categories	Metagenomic sequencing with ViroCap enrichment	NanoString nCounter with 4 cycles of amplification
Sample preparation time	8 to 16 hours for library preparation and assessment 80 hours for targeted sequence capture	1 hour PCR amplification
Data generation time	44 hours on the Illumina NovaSeq 6000 S4 Flowcell	27.5 hours on the NanoString nCounter Flex
Data analysis time	6 to >48 hours	<1 hour
Cost for reagents	~$100/sample (in optimal batch sizes)	~$116/sample

## DISCUSSION

In this study, we demonstrate the use of a targeted, multiplexed molecular assay in capturing key features of sequencing assays for the characterization of vaginal bacterial and viral communities. We found that the custom NanoString nCounter platform improved the cost and time required for the assay, compared to those of sequencing. For bacteria, detection and relative abundance were similar for both assays. For viruses, sequencing detected more low abundance targeted species due to the enhanced detection afforded by a ViroCap enrichment. The NanoString nCounter assay for vaginal microbes is a good option for the highly multiplexed screening of many samples for specific taxa or community characteristics (e.g., approximations of relative abundance and diversity).

We compared the species-level taxonomic classifications of the nCounter and of metagenomic shotgun sequencing (MSS) in this study; however, many studies use 16S rRNA gene sequencing to characterize bacterial communities at the genus level. The nCounter results for species within the same genus could be combined to generate an approximation of a genus-level result. This would be comparable to 16S rRNA gene sequencing results, provided that each of the species was represented in the nCounter probe set. Alternatively, nCounter probes could easily be designed to target genome sequences that are conserved within a genus if genus-level detection is suitable for the goals of a study.

Although agreement on bacterial relative abundance was high overall, there were some discrepancies. For example, *Sneathia vaginalis* was more prevalent in the nCounter assay than in the MSS results. In the case of bacterial analysis, nCounter tended to perform better than sequencing. The discrepancies are likely explained by two factors. First, some taxa have a limited number of reference genomes available. As more genomic diversity is represented in the reference genome database by the addition of sequenced genomes, MSS classification improves (19). This is likely the explanation for the discrepancy in *Sneathia vaginalis* prevalence. Second, many discrepancies are found in microbes with a low signal in either the sequencing or nCounter assay, suggesting that the microbes are present at a low abundance. Discrepancies in these cases are not surprising, as low abundance microbes are likely to differ in their detection, even in replicates of the same assay. As the nCounter assay tended to perform better for low abundance bacteria, sequencing detection could be improved either by deeper sequencing or by adding a targeted enrichment step for bacteria, as we have done for viruses.

Agreement between the two assays was found for just under half of the viruses detected. However, more viruses were detected by the highly sensitive ViroCap enrichment and sequencing assays than were detected by the nCounter assay. One likely explanation of this is that the NanoString nCounter assay did not consistently detect copy numbers of less than 10 per reaction. Sequencing could have an advantage for detecting viruses with larger genomes because there are many different fragments spanning the genome that could be captured and sequenced from each viral genome copy, whereas the nCounter is limited to the detection of 3 specific probe-binding regions. This is consistent with the observation that two viruses with relatively large genomes (cytomegalovirus and molluscum contagiosum) were detected only with sequencing. In 5 instances, a virus was detected by nCounter but not by sequencing. This could also be explained by the detection of low abundance microbes, which can be detected by chance if they are near or below the limit of detection. The detection of low abundance microbes might be improved for the nCounter panel in future assays by adding more amplification cycles before assessing the samples. In this study, we found that the over-amplification of abundant microbes can saturate the signal on the nCounter. Therefore, one could consider not amplifying those microbes that are typically prevalent (e.g., *Lactobacilli*) prior to detection.

Overall, the financial and time costs were lower with the NanoString nCounter assay compared to sequencing with ViroCap. Sequencing assays are relatively expensive, despite the many innovations that have reduced their costs. For microbial community characterization (bacteria, viruses, eukaryotic microbes), we spend ~$100 per sample for sequencing reagents when running optimally sized batches on an Illumina NovaSeq instrument. The NanoString nCounter assay costs about the same as sequencing in terms of reagents, but it involves substantially less costly personnel time. While the amount of hands-on time required can vary with the protocols selected, sequencing with targeted sequence capture enrichment can exceed 10 times the hands-on personnel time required for the nCounter assay. The time to result is also shorter with the nCounter assay compared to that of sequencing. Sequencing assays can be time-consuming (multiple days to >1 week), as they include sample assessment, library construction, target enrichment (in some cases), and, finally, sequencing. The data analysis for Illumina sequencing, with its short sequence read length, is also more complex, which adds to the time required for results. Comparatively, the nCounter assay simply requires a 4-cycle amplification, an overnight hybridization, loading on the nCounter Prep Station, followed by the reading of the counts on the nCounter Digital Analyzer. These data require minimal formatting in the form of background subtraction prior to reporting. While sequencing assays have broad utility and give us the power to thoroughly study microbial genes and genomes, efficient multiplex assays enable us to screen many samples to look for specific patterns correlated with a disease and to test hypotheses related to the characteristics of common microbial features.

This proof-of-concept study demonstrates great promise for the rapid characterization of vaginal microbial communities with a multiplex assay. The strengths of this study include the use of both a mock microbial community and clinically collected vaginal swabs from patients. Another strength is the direct comparison of the targeted assay to the more comprehensive sequencing results in the patient samples. A weakness of this study is the proof-of-concept nature of the panel, which was built to include only 30 preselected microbes that are known to be in the vaginal microbiome.

This work builds upon the many previously reported sequencing-based analyses of the vaginal microbiome and paves the way for streamlined clinical assessment for research studies in the short term and for clinical diagnosis or risk stratification in the long term. The assay described here is a compromise between other common assays, marrying the ease of targeted molecular assays with the highly multiplexed microbe detection found with sequencing.

Future directions should include the further optimization of the assay to detect lower abundance microbes and viruses. We could increase the number of cycles of amplification for viruses and other low abundance microbes. Future studies should also explore the expansion of the current panel. With the expansion of the panel, additional testing for probe specificity will be required to ensure the ability to distinguish similar species (e.g., *Gardnerella* or *Prevotella* species). One might also consider the tailoring of the targets to assess specific sets of microbial targets that are associated with specific diseases. For example, a comprehensive set of the microbes targeted in commercial assays for bacterial vaginosis or vaginitis could be included in a revised panel, and the nCounter panel could then be directly compared to those commercial tests (20). While this assay was developed to target vaginal microbes, the same approach could be applied to rapidly assess subsets of taxa in well-characterized, complex microbial communities. For example, as we learn more about the features of bacterial communities that associate with clinical disease, panels could be built to assess specific taxa, relationships between taxa, or dynamic changes in taxa that are relevant to the prediction or diagnosis of disease (20–22). The nCounter system is extensible to up to 800 probes. So, theoretically, panels could be defined to target 266 to 800 microbial targets (depending on the number of probes used to target each taxon). While the nCounter panels are not as expansive as sequencing assays and will not replace sequencing entirely, they would allow for the rapid screening of relatively complex constellations of taxa within samples, which could allow us to potentially identify fewer samples to carry forward to more complicated and time-consuming analyses.

## MATERIALS AND METHODS

### Samples.

We used a mock community for the initial NanoString nCounter platform testing. This sample was composed of a lysate of herpes simplex virus type 2 (HSV-2) cultured in Vero cells mixed with the Vaginal Microbiome Genomic Mix (ATCC, Manassas, VA), which contains Gardnerella vaginalis, Lactobacillus gasseri, Mycoplasma hominis, Prevotella bivia, Streptococcus agalactiae, and Lactobacillus jensenii in equal copy numbers.

43 vaginal swab samples from human subjects were obtained from the Women and Infants Health Specimen Consortium Biobank (https://womeninfantsbank.wustl.edu/biobank-services/). Samples were not selected based on clinical criteria. Informed consent was obtained, and protocols were approved by the Human Research Protection Office at the Washington University School of Medicine.

### Nucleic acid extraction from vaginal swabs.

The DNA from the vaginal swabs was extracted using the QIAamp BiOstic Bacteremia DNA Kit (Qiagen, Hilden, Germany). The manufacturer’s protocol was followed with minimal changes. 450 μL of warmed Solution MBL was added to the 2 mL PowerBead Tube. The vaginal swab was then swirled in the PowerBead Tube for 30 s and removed by twirling the swab cotton on the inside of the tube to remove as much of the Solution MBL as was possible. The protocol continued with the heating of the PowerBead Tube at 70°C for 15 min and no additional changes.

### Nanostring nCounter assays.

A set of 30 vaginal microbes was selected for inclusion in a custom NanoString nCounter panel (NanoString, Seattle, WA). The bioinformatics team at NanoString designed PCR primers for amplification and probes to target unique regions from 3 different genes in each of the microbial genomes (Table S1, Table S2). The process for the design and synthesis of a new panel takes about 4 weeks. The mock community and the clinical samples were prepared in two ways for the NanoString nCounter analysis, according to the manufacturer’s instructions. First, in cases in which mock community DNA was assessed without amplification, DNA from the Vaginal Microbiome Mix was sheared to 200 to 300 base pairs using a Qsonica sonicator, and the mix was then diluted from 10^2^ to 10^6^ copies per 10 μL in a diluent of sheared DNA from HSV-2 infected Vero Lysate (30 ng/μL). The diluted microbial DNA (7 μL) was denatured at 95°C for 2 min, incubated on ice for 2 min, and then stored at −20°C until it was tested on the NanoString nCounter instrument, using the nCounter Standard Kit (NanoString, Seattle, WA). In the cases in which the mock community DNA was assessed with 8 cycles of amplification, the DNA was not sheared, and the dilutions were carried out in the same fashion as described above. Vaginal swab DNA was consistently assessed without shearing and with 4 cycles of amplification. For both the mock community and the clinical samples, 5 μL of DNA was amplified according to the manufacturer’s protocol. After the amplification, the DNA was denatured at 95°C for 2 min, incubated on ice for 2 min, and then stored at −20°C until it was tested on the NanoString nCounter instrument, using the nCounter Low Input Kit (NanoString, Seattle, WA). The background signal was defined as the mean plus two standard deviations of the negative-control probes, and the background was subtracted before generating figures and comparing the data with the sequencing results. In order to consider a microbe detected, all 3 probes were required to be positive above the background threshold.

10.1128/msphere.00197-22.2 TABLE S1 NanoString nCounter probe sequences. Download Table S1, TXT file, 0.02 MB. Copyright © 2022 Wylie et al. 2022 Wylie et al. https://creativecommons.org/licenses/by/4.0/ This content is distributed under the terms of the Creative Commons Attribution 4.0 International license.

10.1128/msphere.00197-22.3 TABLE S2 NanoString nCounter amplification primer sequences. Download Table S2, TXT file, 0.01 MB. Copyright © 2022 Wylie et al. 2022 Wylie et al. https://creativecommons.org/licenses/by/4.0/ This content is distributed under the terms of the Creative Commons Attribution 4.0 International license.

### Metagenomic shotgun sequencing (MSS).

DNA libraries were made using the Lotus DNA Library Prep Kit (Integrated DNA Technologies, Coralville, IA), and the manufacturer’s protocol was followed. The enzymatic fragmentation time was 10 min at 32°C. The libraries were dual indexed using the IDT for Illumina TruSeq DNA UD Indexes Kit (Illumina, San Diego, CA). For the enrichment of viral nucleic acid prior to sequencing, all libraries were pooled and mixed with ViroCap targeted sequence capture enrichment probes as previously described (1, 17, 18). The libraries were sequenced on the Illumina (San Diego, CA) NovaSeq instrument. The data were analyzed to characterize the eukaryotic virome as previously described (1, 23). Bacterial communities were analyzed with KRAKEN2 (24), using the Bacteria database, which is comprised of the complete bacterial genomes in the NCBI RefSeq database, which we further supplemented with draft genomes from taxa that were not represented in RefSeq but were important for the characterization of the vaginal microbiome: *Atopobium*, *Megasphaera*, *Prevotella*, and Lactobacillus iners. The species-level sequence data were manually reviewed during the comparison with the nCounter results. The data are available in the Sequence Read Archive under BioProject PRJNA861800.
